# Editorial: The scale-up and sustainability of digital health interventions in low- and middle-income settings

**DOI:** 10.3389/fdgth.2025.1634223

**Published:** 2025-09-22

**Authors:** Bassey Ebenso, Eve Namisango, Ibukun-Oluwa Abejirinde, Matthew J. Allsop

**Affiliations:** ^1^ Leeds Institute of Health Sciences, University of Leeds, Leeds, United Kingdom; ^2^ African Palliative Care Association, Kampala, Uganda; ^3^ Social & Behavioural Health Sciences Division, University of Toronto, Toronto, Canada

**Keywords:** digital health, LMIC (low and middle income countries), scale-up, sustainability, innovation

**Editorial on the Research Topic**
The scale-up and sustainability of digital health interventions in low- and middle-income settings

## Why scale up and sustainability matter for digital health interventions

There is increasing recognition that digital health solutions can strengthen health systems by expanding coverage of healthcare, enhancing safety and quality of services, and optimising resources to make services affordable for underserved populations (1, 2). The last decade has seen the proliferation of digital health interventions, defined as the use of information and communications technology in support of health and health-related fields. Despite increasing global investment in and sophistication of digital health solutions, reports from low- and middle-income countries (LIMCs) have been skewed towards short-term (12–24 months) pilot interventions, feasibility and acceptability studies (3). There remains uncertainty in the long-term sustainability of most digital health solutions (4). This suggests that the chances of achieving long-term benefits of digital health, such as universal health coverage and health-related Sustainable Development Goals by 2030, remain slim, as most pilot innovations are seldom brought to scale.

Existing literature is beginning to explore and determine multiple factors that can act as both enablers and barriers to scale-up at the micro (e.g., end user characteristics, social support), meso (e.g., regional infrastructure, culture) and macro (e.g., regulation, funding, reimbursement) levels (5). This is happening alongside the identification of areas that require consideration for scale-up, including program characteristics, human factors, technical factors, healthcare ecosystem, and the extrinsic ecosystem (6). But there remains a need for evidence to inform strategies, frameworks and models that can guide scale-up of digital technologies for diverse use cases including, for example, across reproductive, maternal and child healthcare, infectious diseases, chronic care, emergency preparedness and response, and health system integration and interoperability, particularly in low-resource settings.

In recent years, particularly in the context of low-resource settings, the COVID-19 pandemic has triggered escalated development and use of digital health tools and restructuring of the health ecosystem (7). With increasing innovation and digital health solution development, it is critical to develop and expand the evidence base to guide scale-up and sustainability and explore opportunities for its consideration from the earliest stages of intervention design through to large-scale testing and evaluation.

## This research topic

This Research Topic presents multidisciplinary papers that explore and document evidence identifying contextual factors influencing the scale-up and sustainability of digital health solutions, and theoretical or practical strategies for overcoming barriers to scale-up.

In total, eight (8) papers were included in the collection, providing findings from studies conducted across countries in Africa (i.e., Nigeria, Rwanda, Sierra Leone and South Africa). Included papers encompassed a wide range of topic areas including management of COVID-19, sexual and reproductive health and maternal healthcare, specific technology approaches such as the role of artificial intelligence and machine learning, and broader applications of digital technology for health insurance, human resources strengthening, patient identification techniques, tackling misinformation and disinformation, and enhancing universal health coverage.

Across the collection, scale-up has been defined as the embedding of a digital health product into each level of the health system (policy, practices, workflows, and daily lives of health workers) to improve output (access, scope, quality, efficiency), outcome (coverage, utilization) or impact (morbidity or mortality) rather than regrading digital interventions as standalone initiatives. Sustainability is defined as the longevity and continuing manifestation of benefits and outcomes of digital solutions on the health workforce, standard of healthcare, patient experience, and the environment long after the initial phases of implementation of the technology.

The focus on scale-up and sustainability varied from studies that were horizon scanning and considering scale-up during the early stages of intervention development, alongside articles outlining interventions that have been scaled up nationally and reflect on the process and longer-term sustainability, alongside a systematic review of barriers and facilitators for sustainability.

### Focusing on the future in the early stages of development

Castor et al. present work at the earlier stages of intervention development, in preparation for a clinical study in which they compare the quality of images captured by two devices: a commercially available mobile phone and a hand-held colposcope that captures and stores digital photographs of the cervix and can interface with a cloud-based machine-learning algorithm to generate an automated diagnosis based on the cervical image. The study reports that, whilst the technical performance of digital devices is good, there remain several operational considerations that need careful attention to ensure that mobile phone-based cervical cancer screening approaches can operate well in the context of clinical services.

At a similarly earlier phase of development, Chukwu et al. present findings from a literature review and survey of health facilities in the Federal Capital Territory of Nigeria to understand existing approaches used to generate unique patient identifiers and consider opportunities for scalable solutions. The authors present an algorithm for universal offline unique patient identifier generation and provability. The algorithm meets characteristics deemed important by the authors for scalability, which include requiring no central authority, being cryptographically provable, being metadata discoverable, and a patient identifier that can outlive the issuing institution. Such early development work, with a focus on scalability, could lead to an approach that improves care coordination, data privacy and seamless exchange of patient health records.

### Lessons from early stages of rollout

A study focusing on a digital intervention in the early stages of rollout was reported by Udenigwe et al. The team explored gender inequalities in the use of Text4Life technology in two communities in Edo State, Nigeria. Text4Life is designed to enable instant reporting of pregnancy-related events and timely notification of health facilities. The study explored experiences of women, their spouses, and chairpersons of community committees who facilitate links between health facilities and their communities. The study highlights that whilst the intervention made women feel safer during pregnancy, the wider context of mobile phone use by women remains challenging for intervention implementation, including women having less access to phones than men, women being less likely to participate in digital spaces, and often being excluded from the benefits of mobile health approaches especially when programs are designed without any regard for gender, age, ethnicity or disability. Whilst opportunities for overcoming some challenges are suggested in the study (e.g., provision of free phone for pregnant women, the program beneficiaries), the study emphasizes the importance of both men's involvement in maternal health in a gender transformative manner, and the need to elevate women's leadership in overseeing the design and implementation of maternal health programs to give women a voice in determining programs they need but also enabling them to take ownership and ensures the sustainability of programs.

A study outlines lessons from Sierra Leone by Chukwu et al. with the implementation of a large-scale sexual and reproductive health mHealth intervention. The work highlights multiple challenges and facilitators to success but underscores the need for digital health interventions to be context-specific, with continuous evaluation and adaptation to local conditions, which are critical for long-term impact and effective integration into health systems.

A further study outlining the implementation of a digital health solution across multiple regions is reported by Okuzu et al. The team reports on the development and deployment of a bespoke digital health insurance scheme to support the expansion of health insurance coverage, particularly among poor and vulnerable populations across three states in Nigeria. Following the deployment of the digital health solution across multiple states, contextual enablers of adoption and scale-up included a favourable policy environment (e.g., alignment of digital solution with existing national government commitments to health insurance), public-private-partnerships (e.g., the presence and support for such partnerships, also encouraged through the National Health Insurance Authority Act in Nigeria), and sustained stakeholder engagement (e.g., the continuous engagement of National Health Insurance Authority, State Social Health Insurance Agencies and their ICT personnel). The value and need for ongoing stakeholder engagement as a means of achieving the long-term success of digital health interventions arose in an article by Babili et al. Their qualitative study explored the sustainability and scalability of an SMS-based digital health intervention used in Rwanda's home-based care program for COVID-19. Political commitment to digitising the public health response in Rwanda, alongside an advanced digital infrastructure, was seen as a major contributor to their implementation. Furthermore, collaboration between key stakeholders that included private, governmental and non-governmental partners was seen as necessary to develop solutions for addressing barriers to the adoption of digital technology approaches at the national level.

### Commentary on future directions and considerations for scale-up

This collection includes a systematic review by Kaboré et al. focusing on barriers and facilitators for the sustainability of digital health interventions in low and middle-income countries. The review highlights that the sustainability of digital health interventions is shaped by a range of interconnected factors, including infrastructure limitations and the level of stakeholder engagement. While barriers such as inadequate equipment, connectivity, and power persist, key facilitators include strong governmental and institutional commitment, stakeholder collaboration, and user trust and confidence. Otaigbe provides an overview of the potential benefits of artificial intelligence (AI) to inform healthcare delivery across Africa, including for clinician decision making, early case detection, and diagnosis. However, whilst there could be multiple applications, there remain very few national strategies for operationalising AI, a lack of technical expertise, infrastructure deficits, and high costs of AI deployment. Otaigbe underlines that many of the challenges highlighted can be overcome to enable further exploration of the application of AI across Africa.

## Summary and future directions

The evidence we present within this collection is multifaceted, encompassing diverse research types and providing multiple first-hand accounts of differing levels of implementation, drawing out key factors to consider in planning for and assessing their potential for scale-up and sustainability. Across the articles included in this collection, there is evidence of multiple best practices around scaling digital health, including designing with the end user, building for scale and sustainability, addressing privacy, and being collaborative (6). Broadly across digital health, there is more evidence required to guide the implementation and evaluation of solutions to inform decisions around scale-up and move towards universal coverage (8).

The COVID-19 pandemic undoubtedly heightened the importance of digital health technologies and their application as part of care delivery, providing routes to guaranteeing access to services for millions who could not obtain in-person care. Whilst digital health provides the potential to remove barriers to health care and tackle established global health inequities for vulnerable groups, a global post-pandemic vision for digital health remains unclear, with a need for country and context-specific adaptation of approaches to its use (9). Multiple, established approaches to user involvement can be utilised to support the design and adaptation of digital health interventions for specific contexts (10). This is supported by examples of approaches that map and determine user preferences and needs for digital technologies applied to specific disease groups across multiple stakeholder groups to guide subsequent technology development (11).

A critical area moving forward is the need for greater understanding around digital health equity, ensuring conscious design of approaches to ensure they meet the needs of the most deprived, to avoid exclusion (12, 13). Work by Okuzu et al. provides evidence that digital technology can be used to increase access to, for example, health insurance in vulnerable populations where requisite enablers are in place (e.g., key stakeholder engagement and a supportive policy environment to which the intervention aims are aligned). However, Udenigwe et al. highlight remaining challenges around gender and cultural inequalities in digital health. Two areas of critical questioning remain if digital health is to evolve to produce scalable and sustainable tools to support universal health coverage. First, how can scaling digital health interventions promote equity and inclusion? The World Health Organization (14) recently recognised health equity as one of four cardinal principles of its Global Strategy on Digital Health (2020–2025), requiring investment in infrastructure, education, and resources to help LMICs adopt and scale novel digital health interventions. Second, what difference has the global rapid increase in the application of digital health approaches during and after COVID made in the lives of patients? Has it had an impact on universal health coverage as part of SDG-3, and to what extent has it closed the health inequity gap for different groups (youths, elderly patients, mental health patients)?
